# A compound-target pairs dataset: differences between drugs, clinical candidates and other bioactive compounds

**DOI:** 10.1038/s41597-024-03582-9

**Published:** 2024-10-21

**Authors:** A. Lina Heinzke, Barbara Zdrazil, Paul D. Leeson, Robert J. Young, Axel Pahl, Herbert Waldmann, Andrew R. Leach

**Affiliations:** 1 European Molecular Biology Laboratory, European Bioinformatics Institute, Wellcome Genome Campus, Hinxton, Cambridgeshire CB10 1SD United Kingdom; 2 Paul Leeson Consulting Ltd, Nuneaton, Warwickshire CV13 6LZ United Kingdom; 3 Blue Burgundy Ltd, Ampthill, Bedfordshire MK45 2AD United Kingdom; 4 Compound Management and Screening Center, Max-Planck-Institute of Molecular Physiology, Otto-Hahn-Str. 11, 44227 Dortmund, Germany; 5 Department of Chemical Biology, Max-Planck-Institute of Molecular Physiology, Otto-Hahn-Straße 11, 44227 Dortmund, Germany; 6 Faculty of Chemistry and Chemical Biology, Technical University Dortmund, Otto-Hahn-Straße 6, 44227 Dortmund, Germany

**Keywords:** Cheminformatics, Chemical libraries, Cheminformatics, Chemical libraries, Drug discovery and development

## Abstract

Providing a better understanding of what makes a compound a successful drug candidate is crucial for reducing the high attrition rates in drug discovery. Analyses of the differences between active compounds, clinical candidates and drugs require high-quality datasets. However, most datasets of drug discovery programs are not openly available. This work introduces a dataset of compound-target pairs extracted from the open-source bioactivity database ChEMBL (release 32). Compound-target pairs in the dataset either have at least one measured activity or are part of the manually curated set of known interactions in ChEMBL. Known interactions between drugs or clinical candidates and targets are specifically annotated to facilitate analyses of differences between drugs, clinical candidates, and other active compounds. In total, the dataset comprises 614,594 compound-target pairs, 5,109 (3,932) of which are known interactions between drugs (clinical candidates) and targets. The extraction is performed in an automated manner and fully reproducible. We are providing not only the datasets but also the code to rerun the analyses with other ChEMBL releases.

## Background & Summary

Understanding the reasons a compound succeeds or fails during the drug discovery process is a complex problem. Despite numerous approaches to reduce the number of failures, attrition rates and R&D costs in drug discovery remain high^1–3^. One major obstacle in retrospective analyses of the drug discovery process is the limited availability of high-quality open-source data spanning different stages of the drug discovery pipeline, including compound bioactivity data from the preclinical and clinical phases, as well as data about approved drugs. The use of company data is limited to occasional collaborations between pharmaceutical companies^2^ and analyses of in-house data.

One of the main resources for open-source bioactivity data is ChEMBL^4^. Bioactivity data in ChEMBL covers a broad range of different compounds, bioactivity endpoints, assays, targets, and organisms. In addition, ChEMBL provides data for all stages of the drug discovery process: patent bioactivity data; preclinical compound data from literature and donated by collaborators; data on clinical candidates, including information on their highest clinical phase (MAX_PHASE); as well as drug data with annotations to indications and drug warning information. The dataset presented here extracts pairs of interacting compounds and targets from ChEMBL for which there are measured activities or which are in a table of manually curated disease-relevant interactions in ChEMBL (DRUG_MECHANISM table). Various compound and target annotations are added to facilitate analyses of sets of compounds that interact with the same target or a target in the same target class. A similar dataset was curated previously to identify differences in drug-like properties and ligand efficiencies between drugs and comparator compounds binding to the same target^5^. The herein presented work has extended the previous dataset to include clinical candidates and newer ChEMBL data. Furthermore, the dataset can now be generated in a fully reproducible and automated manner for every ChEMBL version from ChEMBL 26 onwards. As with all databases, the data in ChEMBL are not complete. The bioactivity data and related compounds, targets, and assays that people choose to publish represent certain areas of scientific interest and are often biased towards positive findings^6^. New research might cover other areas of research foci and sometimes uncover inaccuracies in earlier scientific findings. We still hope that the automatic generation of the dataset will allow the exploration of the status quo as knowledge advances.

The dataset in this study was generated from ChEMBL 32. Table 1 provides an overview of the numbers of compounds, targets, and compound-target pairs for the full dataset and for one of the available subsets (*BF_100_c_dt_d_dt)* of the dataset. The subset is limited to targets with at least one hundred compounds with a measured activity at that target and at least one drug or clinical candidate that is known to interact with the target. These criteria limit the subset to targets with enough data for which a comparison of drugs and clinical candidates with other compounds is possible, i.e., targets which are particularly interesting for exploring drug discovery-related questions. However, the full dataset is also made available and has no restrictions on the number of compounds, drugs or clinical candidates per target. The BF_100_c_dt_d_dt subset illustrates one of the available filtered subsets of the dataset that might be of particular interest. The number of compound-target pairs and compounds is similar for both the full dataset and the subset. However, the number of targets in the subset is less than half of the number of targets in the full dataset. This implies that the filtering criteria remove a significant number of targets with a small number of compounds from the dataset, respectively. In total, the dataset (subset) comprises 614,594 (583,398) compound-target pairs with 5,109 (2,639) drugs and 3,932 (2,619) clinical candidates that are known to interact with the respective target.

**Table 1:** Number of compound-target pairs, compounds and targets for the full dataset and the subset BF_100_c_dt_d_dt. The subset BF_100_c_dt_d_dt only includes targets with at least one hundred active compounds and at least one drug or clinical candidate known to interact with the target.

	Total		Comparator compounds		Drugs		Clinical candidates	
	*all*	*BF_100_c_dt_d_dt*	*all*	*BF_100_c_dt_d_dt*	*all*	*BF_100_c_dt_d_dt*	*all*	*BF_100_c_dt_d_dt*
# compound-target pairs	614,594	583,398	605,553	578,140	5,109	2,639	3,932	2,619
# compound-target pairs (incl. variant targets)	624,989	588,120	615,077	582,684	5,623	2,743	4,289	2,693
# compounds	402,282	384,450	400,167	382,727	1,740	1,328	1,578	1,403
# targets	1,398	605	1,117	605	845	383	945	544
# targets (incl. variant targets)	2,287	629	1,943	629	1,057	405	1,138	564

The number of compound-target pairs and targets is given with and without counting targets with different mutations as separate targets. Each number is given based on all compound-target pairs (total) as well as based on pairs for which the compound is marked as a drug, a clinical candidate or neither (a comparator compound) known to interact with the target.

**Fig. 1:**
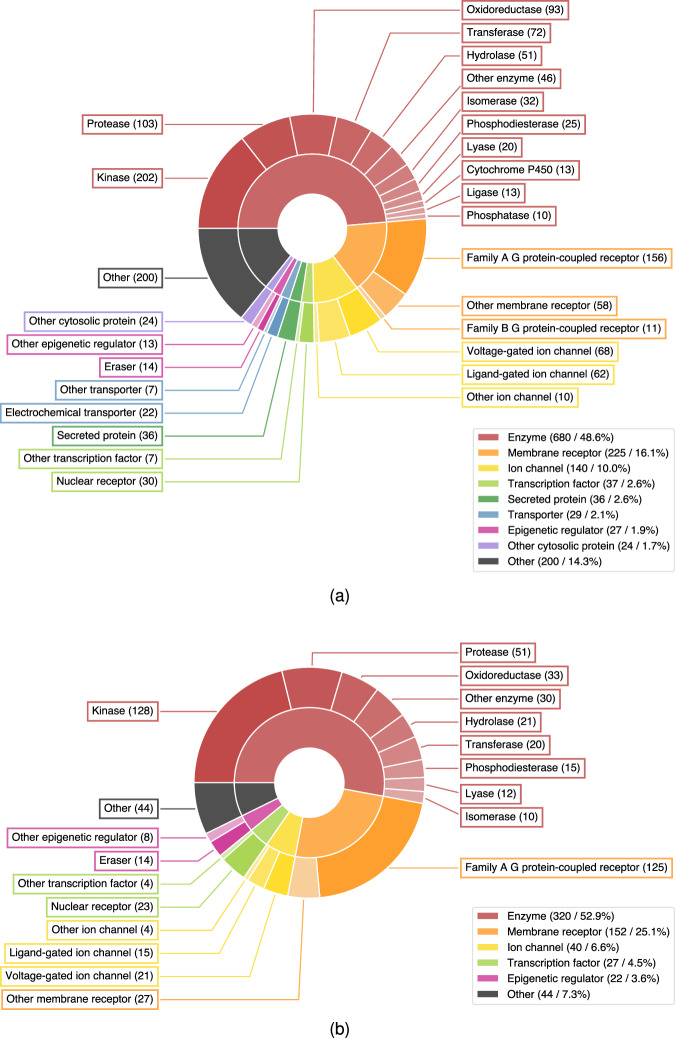
Distribution of target classes in the full dataset (**a**) and in the BF_100_c_dt_d_dt subset (**b**). The inner circle of the pie chart shows the distribution of the more general level 1 target class description in ChEMBL, while the outer circle shows the distribution of the more detailed level 2 target class description in ChEMBL. Targets with more than one target class description and targets with the description ‘Unclassified protein’ are grouped into ‘Other’. Smaller level 1 (level 2) target classes with less than twenty (ten) targets are displayed as ‘Other’ as well.

The dataset contains information about a wide variety of different targets and target classes. The distribution of target classes in the dataset and the *BF_100_c_dt_d_dt* subset are shown in Fig. 1. In both cases, about half of the targets in the dataset are enzymes, with kinases being the most common enzyme class. This is followed by membrane receptors, mainly comprising family A GPCRs, and making up 16.1% and 25.1% of the full dataset and the subset, respectively. Other noticeable target classes include ion channels and transcription factors which each represent ten percent or less of the targets in the dataset.

## Methods

The workflow to calculate the dataset based on information from ChEMBL consists of three main steps:

1. Query ChEMBL to obtain all relevant compound-target pairs.
2. Add compound and target annotations to each pair and clean the dataset.
3. Extract potentially interesting subsets of the dataset and add filtering columns to the full dataset for easy retrieval of the subsets.

**Fig. 2:**
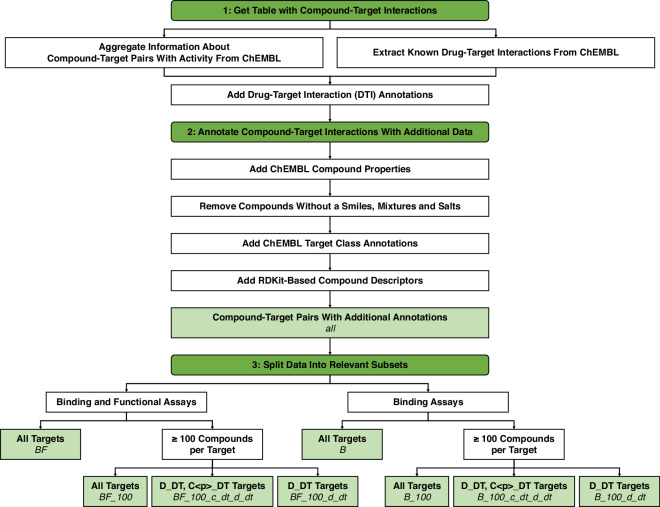
Overview of the workflow used to generate the full dataset and its subsets. The three main steps of the workflow are coloured in dark green. The final dataset and all possible subsets are coloured in light green. The respective file names are indicated in italics.

The steps are outlined in Fig. 2 and explained in detail below.

### Compound-target pairs

The first set of compound-target pairs is obtained from the ACTIVITIES and ASSAYS table. A compound is considered active on a target if it has a pChEMBL value measured in a binding (B) assay (data measuring binding of a compound to a molecular target, e.g., Ki, IC50, Kd) or functional (F) assay (data measuring the biological effect of a compound, e.g., % cell death in a cell line, rat weight). ChEMBL provides pChEMBL values, i.e., the negative logarithmic representation of the molar activity values, for selected concentration-response activity values (IC50, EC50, XC50, AC50, Ki, Kd, potency).

All compounds are mapped to their parent compound through the MOLECULE_HIERARCHY table. The information about the parent compound is subsequently used to identify the compound, and the information about the salt form is dropped. If there is more than one activity measurement for a compound-target pair, the pChEMBL values are aggregated into mean, median and maximum pChEMBL values. The aggregation of pChEMBL values incorporates data from different assay types. Experimental uncertainty has been shown for both IC50 values as well as Ki values^7,8^. While mixing data from different assay types and different labs can be necessary for large-scale analyses^7^, we advise caution when using the aggregated pChEMBL values and pChEMBL-derived values, i.e., ligand efficiency metrics.

Additionally, the publication year in DOCS is aggregated into the year of the first publication of the compound-target pair and the year of the first publication that is associated with a pChEMBL value. All aggregated values are calculated once based on information from binding and functional assays combined (suffix ‘_BF’) and once based on only binding assays (suffix ‘_B’).

The second set of compound-target pairs is obtained from the DRUG_MECHANISM table. The table contains information about the mechanism of action of drugs and clinical candidates and is manually curated based on various sources (e.g., ATC, FDA, ClinicalTrials.gov). Only entries with a DISEASE_EFFICACY (flag to show whether the target assigned is believed to play a role in the efficacy of the drug in the indication(s) for which it is approved) of 1 are taken into account.

Target IDs in the table are mapped to related target IDs to increase the number of target IDs for which there is data in the DRUG_MECHANISM table. Both the original as well as the mapped target IDs are kept. The mapping is based on a subset of the mappings in the TARGET_RELATIONS table. The subset of considered mappings is shown in Table 2. For example, a target ID of a protein family is mapped to the target IDs of all the single proteins that belong to the target family.

**Table 2:** Types of target relations in ChEMBL that are used to map target IDs to related target IDs.

Original target type	Target relationship	Related target type
protein family	-[superset of]->	single protein
protein complex	-[superset of]->	single protein
protein complex group	-[superset of]->	single protein
single protein	-[equivalent to]->	single protein
chimeric protein	-[superset of]->	single protein
protein-protein interaction	-[superset of]->	single protein

The original target type is the target type of the target that is to be mapped to a related target, and the related target type is the type of the related target.

Since the DRUG_MECHANISM table only includes known interactions between compounds and targets, the compound-target pairs are not required to have an associated pChEMBL value. Compound-target pairs that are not yet present in the dataset because of a measured activity are added.

Each compound-target pair is assigned a drug-target interaction type (DTI). The different interaction types are shown in Table 3. If the compound-target pair is in the DRUG_MECHANISM table, it is considered to be a known and relevant compound-target interaction. The pairs are annotated as D_DT (drugs) or C<p>_DT (clinical candidates) based on the maximum clinical phase <p> that the compound reached. The remaining pairs are annotated with DT if the target is in the DRUG_MECHANISM table, i.e., if the target plays a role in the disease efficacy of at least one compound, and with NDT otherwise. DT compound-target pairs are kept as ‘comparator’ compounds. Note that these may include approved drugs and clinical candidates when they are approved for another target, but the mechanism of action with the given target is unknown. All NDT pairs are discarded and do not appear in the final dataset.

**Table 3:** Strategy to assign drug-target interaction types (DTI).

In DRUG_MECHANISM table?	max_phase?	Therapeutic target?	DTI annotation	Explanation
Yes	4	—	D_DT	Drug – drug target
Yes	3	—	C3_DT	Clinical candidate in phase 3 – drug target
Yes	2	—	C2_DT	Clinical candidate in phase 2 – drug target
Yes	1	—	C1_DT	Clinical candidate in phase 1 – drug target
Yes	<1	—	C0_DT	Compound in unknown clinical phase – drug target
No	—	Yes	DT	Drug target
No	—	No	NDT	Not drug target

A max_phase of <1 refers to max_phase = 0 in ChEMBL 31 and earlier versions. Since ChEMBL 32, it refers to compounds in phase 0.5 (early phase one), −1 (clinical phase unknown) and NULL (preclinical compounds).

### Compound and target annotations

ChEMBL-based compound properties are added to each compound-target pair. This includes the first publication date of the compound, compound properties from the COMPOUND_PROPERTIES table and compound structures (InChI, InChI key and canonical SMILES). Compounds without a SMILES and compounds with a SMILES containing a full stop, e.g., mixtures, are removed. Since compounds are always mapped to their parents, only a small portion of compounds fit these criteria (2,694 compounds without a SMILES and 273 compounds with a SMILES containing a full stop). Ligand efficiency metrics (LE, BEI, SEI, and LLE) are calculated for pChEMBL values based on binding and functional data (suffix ‘_BF’) and based on only binding data (suffix ‘_B’). First-level ATC classifications are collected for each compound from the MOLECULE_ATC_CLASSIFICATION table and concatenated alphabetically into one descriptor with ‘|’ as a separator.

Two levels of target classes are taken from the PROTEIN_CLASSIFICATION table for each target. Level 1 target classes are more general, e.g., Enzyme, while level 2 target classes are more specific, e.g., Kinase. If a target has more than one level 1 or level 2 assignment, the assignments are concatenated alphabetically with ‘|’ as a separator. Instances with concatenated target class descriptions are written to an output file which could be used to reassign these target classes by hand. In total, there are fifty targets with more than one target class assignment for either level 1 or level 2, some of which have more than one target class assignment for both level 1 and level 2. There are forty-one targets with more than one level 1 target class and twenty-two targets with more than one level 2 target class assignment.

Optionally, RDKit-based^9^ compound properties are calculated and added to the dataset. These include the built-in compound descriptors FractionCSP3 and the number of heteroatoms, stereocenters and various cycles ([aliphatic / aromatic / saturated] [rings / carbocycles / heterocycles]). Furthermore, scaffolds with and without stereo information are added. The number of aromatic atoms, including the total number as well as the number of aromatic carbon, nitrogen, and hetero atoms, are added with a custom RDKit-based function.

### Cleaning and basic checks

Once all annotations are calculated, the dataset is cleaned. Empty strings and numpy.nan values are changed to ‘None’ to ensure consistency. The type of integer columns is explicitly set to Int64. All floating-point values except for MAX_PHASE are rounded to four decimal places.

During the calculations and after cleaning the dataset, a set of basic checks is performed to ensure its consistency. It is checked that all ‘None’ values are properly recognised as such and object-type columns do not contain other types, such as integers. If a compound-target pair does not have a pChEMBL value, it is checked that the pair is in the DRUG_MECHANISM table. The numbers of NULL values for ligand efficiencies, ChEMBL- and RDKit-compound properties, ATC levels and target class annotations in the dataset are checked against the expected number of NULL values based on the number of missing values in the respective tables.

### Filtering columns

Several subsets of the final dataset are calculated. The different subsets with their respective names are shown in the overview of the workflow in Fig. 2. The first type of subset limits the dataset to targets with at least one hundred compounds with a pChEMBL value. The other types of subsets limit the dataset further to targets with at least one clinical candidate or drug that is known to interact with the target, i.e., compounds with a DTI annotation of ‘D_DT’, ‘C3_DT’, ‘C2_DT’, ‘C1_DT’ or ‘C0_DT’. The subsets are calculated for both binding and functional assay-based values (suffix ‘_BF’) as well as values based on binding assays alone (suffix ‘_B’).

The subsets are added to the full dataset as filtering columns, facilitating easy splits of the full dataset, and can optionally be written to individual files. For all output files, it is checked that writing them to a file was successful by reading the file and verifying that the read data is identical to the calculated data.

### Limiting the dataset to literature data

The dataset extraction can be restricted to only include literature sources. This changes some of the values in the pChEMBL columns and in columns which depend on them, i.e., the ligand efficiency metrics. Values in columns related to the first appearance of the compound or compound-target pairs change as well. Since this restriction changes values in several columns, limiting the dataset to literature sources is not available as a filtering column. Instead, it is a parameter that is set before extracting the dataset.

By default, the dataset is limited to include only literature sources to ensure consistency. ChEMBL is based on a variety of different sources, some of which have not been added on a regular basis. One of these sources is BindingDB^10^. BindingDB curated patent data from 2013 onwards, and data from BindingDB was added to ChEMBL until 2016. Therefore, there is a large amount of patent data for the years 2013–2016 in ChEMBL in comparison to all other years.

**Fig. 3:**
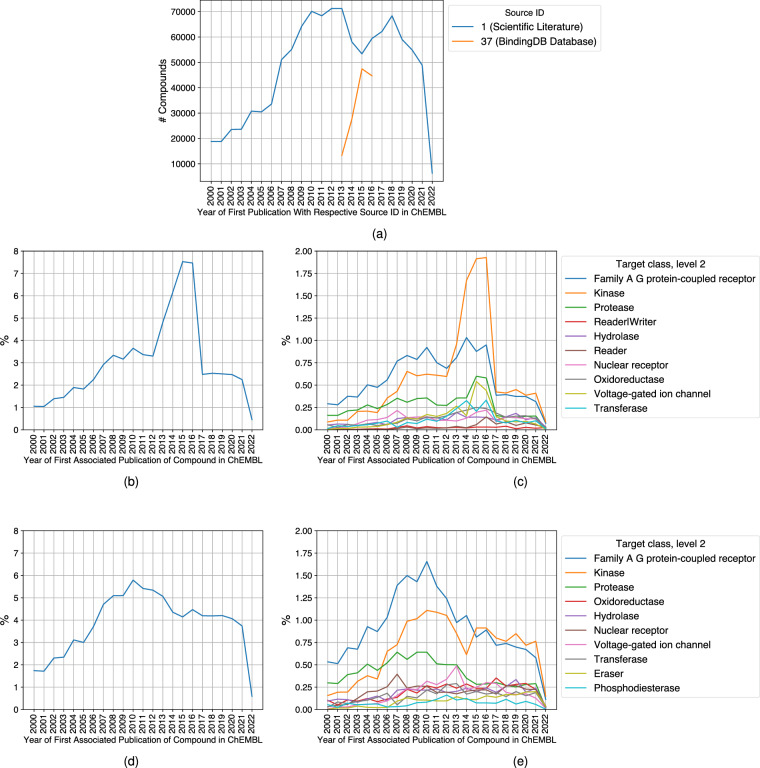
The effects of including literature sources and BindingDB data in the calculation of the dataset. The plots are based on ChEMBL 32 and are limited to the time between 2000 and 2023. (**a**) The number of compounds deposited into ChEMBL from BindingDB and from literature sources by year. (**b**) The percentage of compounds in the dataset first published in a given year when all sources are included. (**c**) The percentage of compounds per one of the ten most frequent target classes first published in a given year when all sources are included. (**d**) The percentage of compounds in the dataset first published in a given year when only literature sources are included. (**e**) The percentage of compounds per one of the ten most frequent target classes first published in a given year when only literature sources are included.

The number of compounds from BindingDB compared to the number of compounds from literature sources can be seen in Fig. 3. When including all sources, the percentage of compounds first published in 2015 and 2016 is more than double the percentage of compounds in the years 2012 and before and after 2016 (Fig. 3(b)). In the years from 2014 to 2016, kinases are overrepresented compared to all other years before and after (Fig. 3(c)). These effects are not seen when the dataset is limited to literature sources (Fig. 3(d,e)). To exclude any effects from this skewed distribution, the default option is set to include only literature data. The option can be changed to include data from all sources in ChEMBL, but we advise caution when using the resulting dataset.

## Data Records

The full dataset and the supporting files were uploaded in CSV format to Zenodo^11^ (10.5281/zenodo.10721939). Additionally, the dataset is available on the ChEMBL FTP site (https://ftp.ebi.ac.uk/pub/databases/chembl/Drug_Target_dataset/). The dataset and its subsets are available for all ChEMBL versions from 26 to 33. For each ChEMBL version, the dataset is available based exclusively on literature sources (‘literature_only’) and based on all available sources (‘all_sources’). All datasets and subsets include the RDKit-based compound properties. Semicolons are used as delimiters in all CSV files. The available files relevant to this work are described below.

### Full dataset

The full dataset is available in the file ‘ChEMBL32_CTI_literature_only_full_dataset.csv’. It includes all compound-target pairs for ChEMBL 32, is based exclusively on literature sources and includes the RDKit-based compound properties. All subsets of the dataset are available to download and can alternatively be obtained from the full dataset by using the filtering columns explained in the documentation in the GitHub repository (see Code Availability). The file names of the subsets and the names of the filtering columns are consistent with the names in Fig. 2.

### Targets with more than one target class assignment

The file ‘ ChEMBL32_CTI_literature_only_targets_w_more_than_one_tclass.csv’ contains all target IDs for which there is more than one level 1 or level 2 target class assignment. The target classes for these targets could be reassigned by hand if one target class per target class level was desirable for the applications of future users. This has not been done for the provided dataset to ensure reproducibility and consistency between different ChEMBL versions.

### Basic dataset statistics

A collection of basic metrics of the full dataset can be found in ‘ChEMBL32_CTI_literature_only_full_dataset_stats.csv’. This includes the numbers of compounds, targets, targets including mutation annotations, compound-target pairs and compound-target pairs including mutation annotations for the whole dataset as well as for drugs, clinical candidates, and comparator compounds. The numbers in the file correspond to the numbers for the full dataset in Table 1. Files named according to the subset names in Fig. 2 and ending in ‘_stats.csv’ provide the equivalent information for the respective subset.

## Technical Validation

All compound-target pairs in the dataset were retrieved from ChEMBL. Most compound-target pairs are included because there exists a specific measured activity in ChEMBL, i.e., a pChEMBL value, for the compound measured on a specific target. pChEMBL values are provided in ChEMBL only if all of the following criteria are met: STANDARD_VALUE must be > 0, DATA_VALIDITY_COMMENT must be NULL or ‘Manually validated’, STANDARD_RELATION must be ‘ = ’, STANDARD_UNIT must be ‘nM’, STANDARD_TYPE must be one of the following: ‘IC50’, ‘XC50’, ‘EC50’, ‘AC50’, ‘Ki’, ‘Kd’, ‘Potency’, ‘ED50’. Duplicates (POTENTIAL_DUPLICATE is not 0), activities with suspected validity problems (DATA_VALIDATY_COMMENT is not NULL) and unchecked targets (TID <  > 22226) are excluded. Preclinical bioactivity data in ChEMBL is extracted from literature sources or imported from other credible sources such as deposited data from neglected disease organisations, project-specific data such as data donated by the Structural Genomics Consortium (SGC)^12^, and data from other databases such as BindingDB^10^. As discussed in the Methods section, the default option for generating the datasets is limited to data from the scientific literature to ensure consistency.

The remaining compound-target pairs that do not possess a pChEMBL value are included because they are listed in the DRUG_MECHANISM table, providing proof for the existence of a therapeutically relevant interaction between the compound and the target. These interactions are “manually assigned using reference sources such as scientific literature, drug package labels and company pipeline information”^13^.

Furthermore, the workflow to calculate the dataset includes several cleaning steps and basic checks, as described in the Methods section, to ensure the reliability of the dataset.

## Usage Notes

The Python code and its documentation can be found in the GitHub repository in Code Availability.

The code can be used by following the installation instructions in the GitHub repository and calling main.py. An explanation of the input parameters is provided when calling ‘python main.py--help’. The full dataset will always be written to a CSV file. Additional outputs and output types can be chosen with the parameters provided in Table 4.

**Table 4:** Available input parameters for the code to generate the dataset.

Parameter	Required	Default	Explanation
--chembl, -v	No	None	ChEMBL version. The latest available ChEMBL version is used if this is not set.
--sqlite, -s	No	None	Path to SQLite database. If this is not set, ChEMBL is downloaded as an SQLite database and handled using the chembl_downloader package.
--output, -o	Yes	—	Path to write the output files to.
--delimiter, -d	No	;	Delimiter in output csv-files.
--all_sources	No	—	Include all sources if this is set. By default, this is not set, and the dataset is calculated based on only literature sources.
--rdkit	No	—	Calculate RDKit-based compound properties if this is set.
--excel	No	—	Write the results to excel. Note: this may fail if the output is too large. The results will always be written to csv.
--BF	No	—	Write the subsets based on binding and functional assays.
--B	No	—	Write the subsets based on binding assays.
--debug	No	—	Log additional debugging information.

Access to ChEMBL is either handled by a given path to a downloaded SQLite ChEMBL database or by the chembl_downloader Python package^14^. Both use SQLite to query ChEMBL.

There have been several changes to the ChEMBL database schema over the different versions, and some of the earlier ChEMBL versions do not include all of the tables or fields necessary to calculate the dataset. Currently, ChEMBL 26 is the earliest version for which the dataset can be calculated.

The documentation for the code is generated automatically with the Sphinx package (https://www.sphinx-doc.org/en/master/index.html) and is linked in the GitHub repository. In addition to the general documentation, it includes a brief introduction, a detailed explanation of the different columns in the final dataset and a short user guide.

## Data Availability

The code used for this work is available on Zenodo (10.5281/zenodo.10723115) and GitHub^15^ (https://github.com/chembl/compound_target_pairs_dataset). The main dataset can be generated with the following call: python main.py --chembl 32 --output <output_path> --rdkit More detailed information on how to use the code can be found in the Usage Notes section.
